# Presence of Natural Killer B Cells in Simian Immunodeficiency Virus-Infected Colon That Have Properties and Functions Similar to Those of Natural Killer Cells and B Cells but Are a Distinct Cell Population

**DOI:** 10.1128/jvi.00235-22

**Published:** 2022-03-21

**Authors:** Andrew Cogswell, Sungro Jo, Natasha Ferguson, Kajal Gupta, Edward Barker

**Affiliations:** a Department of Microbial Pathogens and Immunity, Rush University Medical Center, Chicago, Illinois, USA; Emory University

**Keywords:** B cells, cytokines, cytotoxic, gastrointestinal, gut inflammation, human immunodeficiency virus, immunoglobulin A, mucosal immunity, natural killer cells, simian immunodeficiency virus

## Abstract

Here, we report the appearance of natural killer B (NKB) cells within the colon during simian immunodeficiency virus (SIV) infection of susceptible monkeys. Using RNA sequencing (RNAseq) and flow cytometry, we show that NKB cells are unique cells with features and functions of both NK and B cells. NKB cells express receptors and ligands found on B cells that are important for (i) antigen presentation; (ii) activities associated with class switching, affinity maturation, and B-cell memory formation in secondary lymphoid follicles; and (iii) antigen recognition. The predominant immunoglobulins (Igs) expressed on NKB cells are IgA, although NKB cells can express surface IgM and IgG. There is dominant lambda expression over the kappa light chain characteristic of mucosal B cells. In addition to B-cell aspects, NKB cells express NK cell activation receptors and Fas ligand. We show in this study that NKB cells express perforin and granzymes and lyse cells in a lytic assay. In addition to NK cell cytolytic function, NKB cells also produce the inflammatory cytokines interferon gamma, tumor necrosis factor alpha, and interleukin-18 (IL-18). Finally, we noted the increased capacity of NKB cells to proliferate compared to NK cells and CD8^+^ T cells from the SIV-infected colon. The increased proliferation and inflammatory cytokine production may be related to the relatively high expression levels of IL-15 receptor beta, IL-7 receptor, IL-18 receptor, and 41BB relative to the same receptors on CD8 and NK cells. The properties of NKB cells may point to their role in the enhanced inflammation observed in the SIV-infected gut.

**IMPORTANCE** There is low-level but significant mucosal inflammation in the gastrointestinal tract secondary to human immunodeficiency virus (HIV) infection that has long-term consequences for the infected host. This inflammation most likely originates from the immune response that appears as a consequence of HIV. Here, we show in an animal model of HIV that the chronically SIV-infected gut contains cytotoxic natural killer B cells that produce inflammatory cytokines and proliferate during infection.

## INTRODUCTION

Although the gastrointestinal (GI) tract is critical for the absorption of nutrients and water, it is also reported to be one of the largest (approximately 8.0 m long) human immune organs (1, 2). It contains more B cells (3) than any other tissue in the body and harbors cells involved in the adaptive and innate immune responses (4). Because of its size and exposure to the environment, the gut’s mucosal immune system is under tight regulation so as not to provoke an overactive immune response (5, 6). At the same time, the gut-associated immune system must protect against disease-causing microbes. Thus, the gut immune response must be tightly regulated to quickly react and eliminate enteric pathogens while maintaining general homeostasis when not provoked.

One of the hallmarks of human immunodeficiency virus (HIV)/simian immunodeficiency virus (SIV) infection of the GI tract is a profound loss (>90%) of CD4^+^ T cells within weeks of transmission before a decrease of peripheral blood or nondraining lymph node (LN) CD4^+^ T cells (7, 8). Widespread evidence indicates that T_H_17 cells, a critical T-cell subset in maintaining mucosal homeostasis in the gut, are significantly lost following HIV/SIV infection (9–11). There is a relatively high frequency of HIV-permissive CCR5^+^ CD4^+^ T cells in the GI tract and a highly active state of these cells (8). However, many of the T_H_17 cells are no more susceptible to infection in the gut than other CD4^+^ subsets (9), suggesting that HIV infection is not the direct cause of T_H_17 depletion (12–14). For example, T_H_17 cells could undergo Fas-mediated activation-induced cell death independent of interferon gamma (IFN-γ) (15).

The second aspect of HIV infection of the gut is the appearance of microbial products, particularly lipopolysaccharide (LPS), within mucosal tissue soon after HIV infection (16, 17). The appearance of microbial products in the mucosa is likely the result of the loss of tight junction proteins (TJPs) between the epithelial cells within the protective layer between the lamina propria and the microbes in the lumen of the gut (17). The mechanism leading to this loss of TJPs is not entirely clear. One possible explanation is that TJPs on gut epithelial cells are lost following the gut-associated immune system expression of inflammatory cytokines such as tumor necrosis factor alpha (TNF-α) (18), interleukin-1β (IL-1β) (19), and lymphotoxin-like inducible protein that competes with glycoprotein D for herpes virus entry on T cell (LIGHT) (20). The binding of inflammatory cytokines to their receptors on epithelial cells leads to the increased expression of myosin light chain kinase (MLCK) (19, 21) and the increased activation of MLCK (22). These events are followed by the downmodulation of TJPs (23–25).

HIV infection of the gut is associated with increased mucosal inflammation (16, 26). The immune source of inflammation during infection is extensive, and numerous sources of inflammation have been described (reviewed in reference 27). However, one source of inflammation that has not been explored extensively during HIV/SIV infection of the gut is natural killer B (NKB) cells. NKB cells were initially identified as lymphocytes found in normal mouse spleen and mesenteric lymph nodes (MLNs). NKB cells were unique in that they had markers found on NK cells (i.e., NKp46 and NK1.1) and B cells (i.e., CD19 and IgM) but no other lymphocyte subset-specific markers. Functionally, NKB cells were initially defined as noncytotoxic, unable to secrete IFN-γ, and expressed only IgM. Unlike NK cells and B cells, NKB cells secreted the proinflammatory cytokine IL-18 (28). However, initial studies utilized NKB cells exclusively from the spleen and mesenteric lymph nodes from uninfected mice. Furthermore, even in experiments involving mice infected with GI pathogens, the investigators did not attempt to identify NKB cells in the lamina propria after infection (28). Thus, it remains to be determined if GI pathogens will lead to the recruitment of NKB cells within the lamina propria of the gut.

More recent studies illustrate that NKB (NKG2A/C^+^ CD19^+^) cells can be found in the blood, spleen, MLNs, and GI tissue of uninfected rhesus macaques (29). The NKB cell numbers in the blood and GI tract increase significantly following SIV infection (29). The NKB cells in infected rhesus macaques possess markers (i.e., CD16 and CD40) found on NK and B cells (29). Surface immunoglobulin A (IgA) and IgM were almost universally expressed on NKB cells found within the peripheral blood, spleen, and MLNs of SIV-infected rhesus macaques and HIV-infected humans (29).

Here, we sought to determine the function and characteristics of NKB cells in uninfected and SIV-infected colons and mesocolic LNs of rhesus macaques and cynomolgus macaques. We further evaluated markers found on NK and B cells and determined the unique transcripts present within SIV-infected NKB cells compared with transcripts from B cells and NK cells. We found in our study that NKB cells within SIV-infected colons can kill target cells, proliferate, and express IFN-γ, TNF-α, and IL-18. Our studies thus revealed a probable role of NKB cells in HIV/SIV-associated inflammation.

## RESULTS

### NKB cells are present in the lamina propria of the SIV-infected colon but are not present in the uninfected colon.

**FIG 1:**
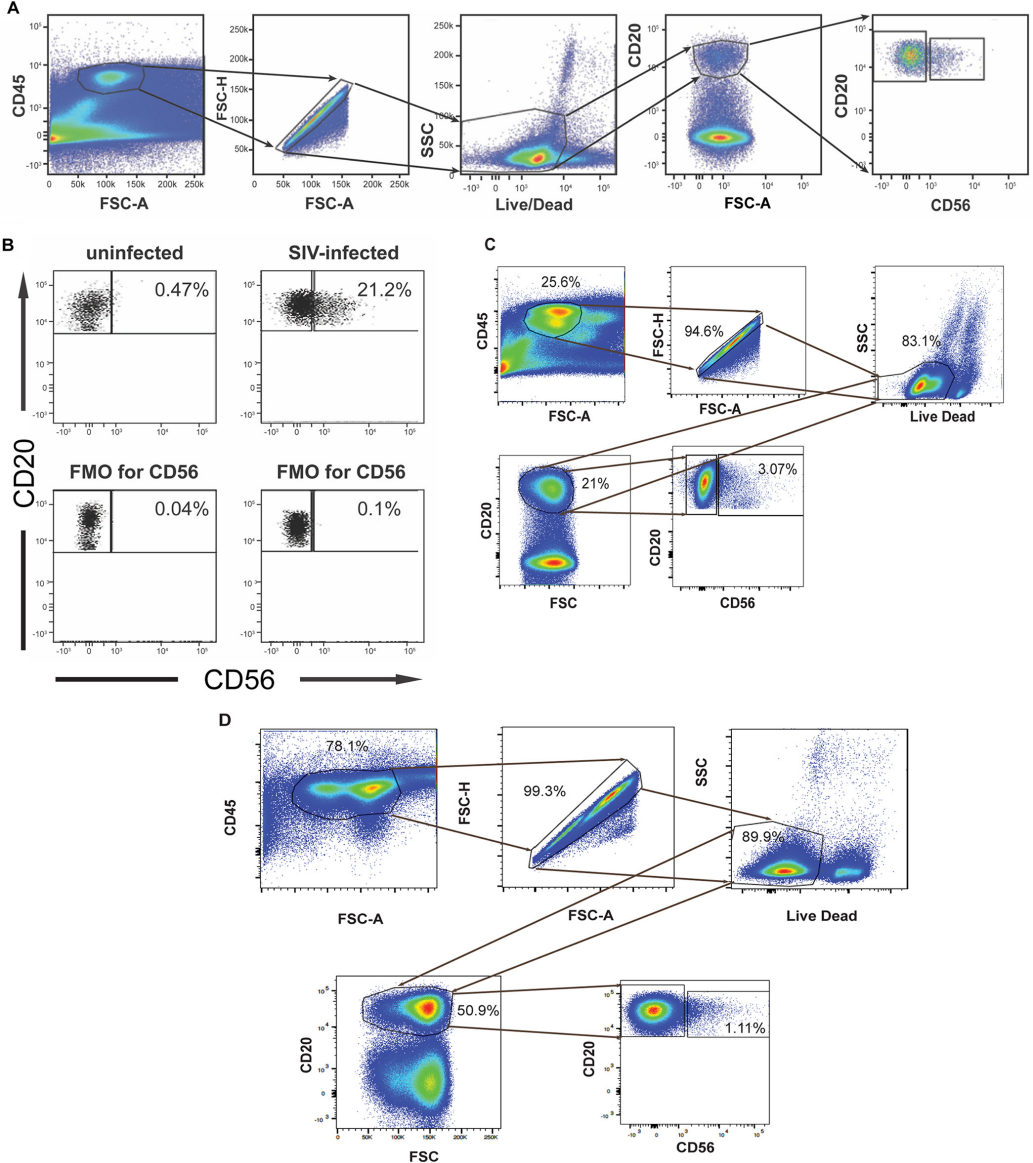
NKB cells are present in SIV-infected but barely present in uninfected colons. (A) The approach used to identify NKB cells by flow cytometry within the colons of SIV-infected macaques. (B) CD45^+^ CD20^+^ CD56^+^ cells within the lamina propria of the colons of uninfected macaques and SIV-infected macaques. (C) CD45^+^ CD20^+^ CD56^+^ cells within acutely SIV-infected colons. (D) CD45^+^ CD20^+^ CD56^+^ cells within SIV-infected mesocolic lymph nodes. FSC, forward scatter; SSC, side scatter; A, area; H, height.

We determined the presence of NKB cells by flow cytometry using thawed liquid nitrogen-frozen cell suspensions from the lamina propria of the SIV-infected colon. The CD20^+^ CD56^+^ viable single leukocytes (CD45^+^) within the stained cell suspension were deemed NKB cells (Fig. 1A; see also Fig. 5A). In the lamina propria of 21 SIV-infected but not 4 uninfected colons of macaques, we found CD56^+^ CD20^+^ lymphocytes in 2 to 10% of the CD20 population (examples are shown in Fig. 1B). NKB cells were also found in acutely SIV-infected colons (30 days postinfection) in four animals, ranging from 2.2% to 5.6% of the CD20 population (Fig. 1C). We also found NKB cells in the SIV-infected draining lymph nodes (LNs) of the colon (i.e., mesocolic LNs) (Fig. 1D). These findings indicate that NKB cells are present in the colon and the colon draining lymph nodes during SIV infection.

### NKB cells share receptors, markers, and functions of NK cells and B cells.

**FIG 2:**
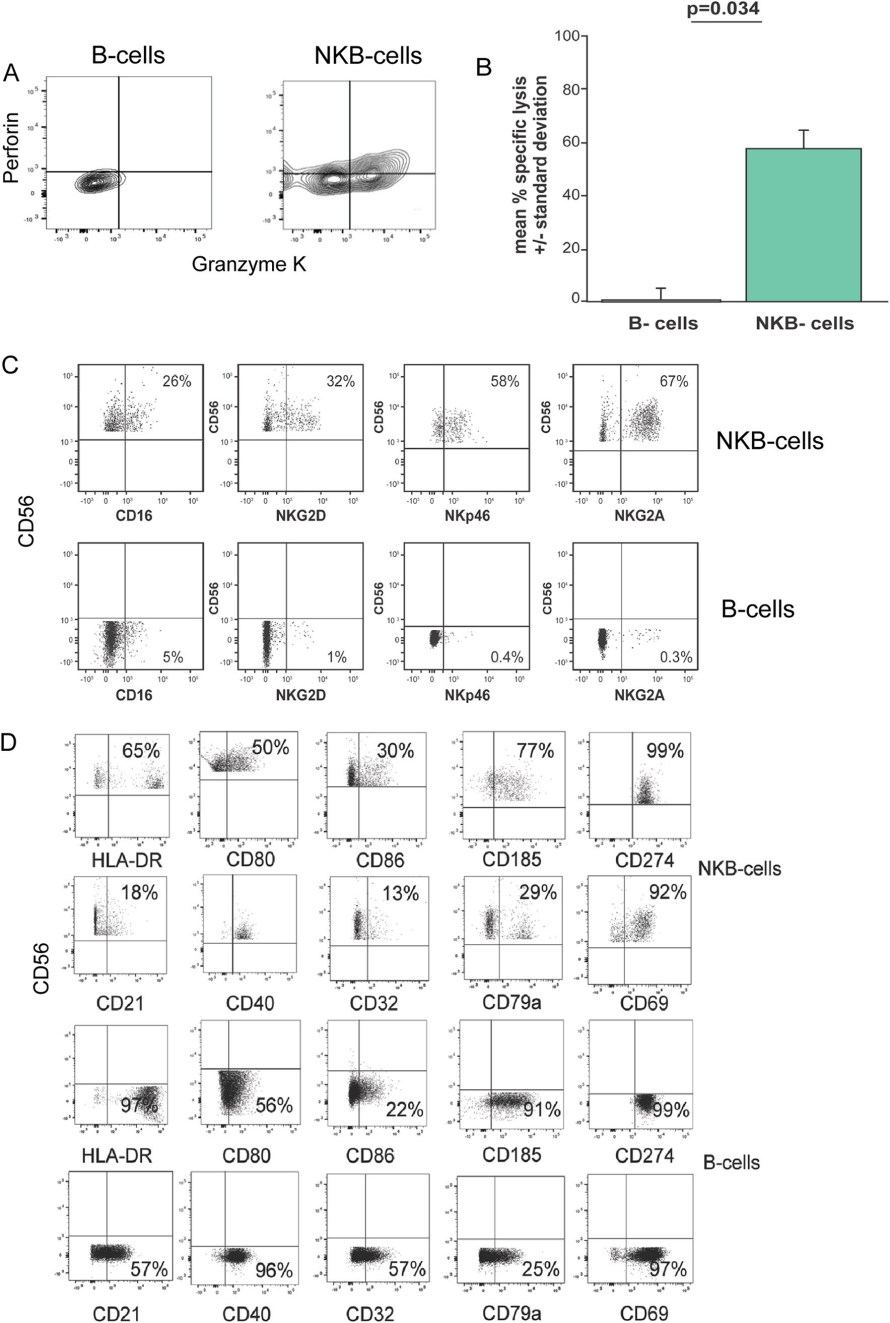
NKB cells in the SIV-infected colon are cytolytic and possess markers and receptors found on NK and B cells. (A) Intracellular staining of perforin and granzyme K within B cells and NKB cells. (B) Mean specific lysis of K562 cells in a 4-h cytolytic assay using B cells and NKB cells as effector cells. Error bars are the standard deviations from three separate wells of target and effector cells at a 1:1 effector-to-target cell ratio. (C) Frequency of NKB cells expressing markers and receptors present on NK cells. Results for NKB cells are shown in the top row, and results for B cells are shown in the bottom row. (D) Frequency of NKB cells expressing markers and receptors typically expressed on B cells. Results for NKB cells are shown in the top two rows. Results for B cells are shown in the bottom two rows.

We found that CD56^+^ CD20^+^ viable single leukocytes (i.e., NKB cells) in SIV-infected colons not only expressed perforin and granzyme K (Fig. 2A) but also were cytotoxic, as shown by their ability to lyse K562 cells, as opposed to B cells that did not (Fig. 2B). NKB cells expressed the NK cell receptors CD16, NKG2D, NKp46, and NKG2A/C (Fig. 2C). In addition to NK cell markers, we noted that these cells expressed B-cell markers (Fig. 2D). These include molecules important for antigen presentation, such as major histocompatibility complex (MHC) class II molecules, CD80, and CD86. In addition, NKB cells express molecules known to be present on B cells, such as CXCR5 (CD185), PDL1 (CD274), CD40, and CD79a. Very few NKB cells express CD21 and CD32b, even though many more B cells express these markers. In addition, we noted that almost all NKB cells express CD69 (Fig. 2D), a typically abundant molecule on lymphocytes within mucosal tissues.

**FIG 3:**
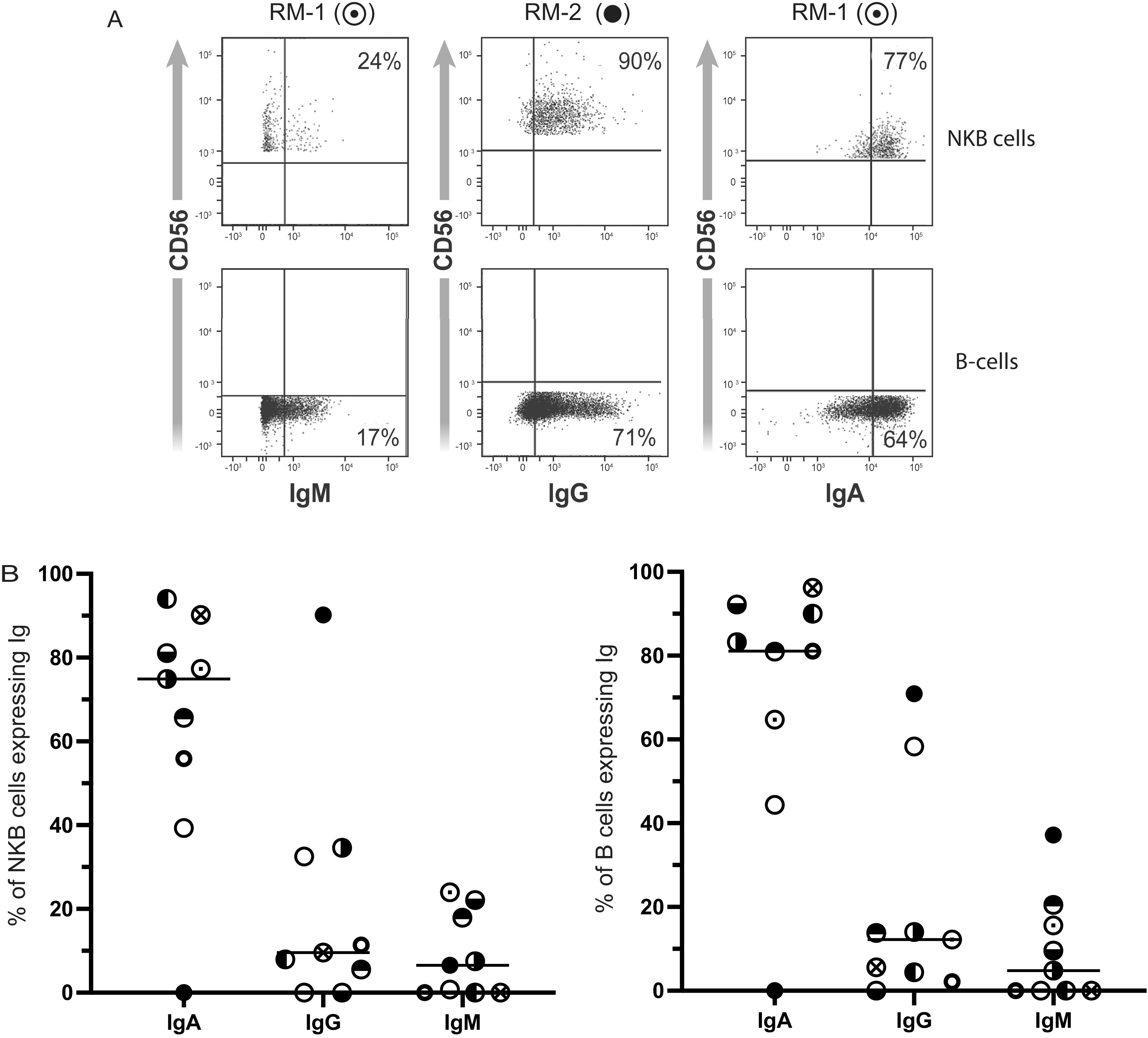

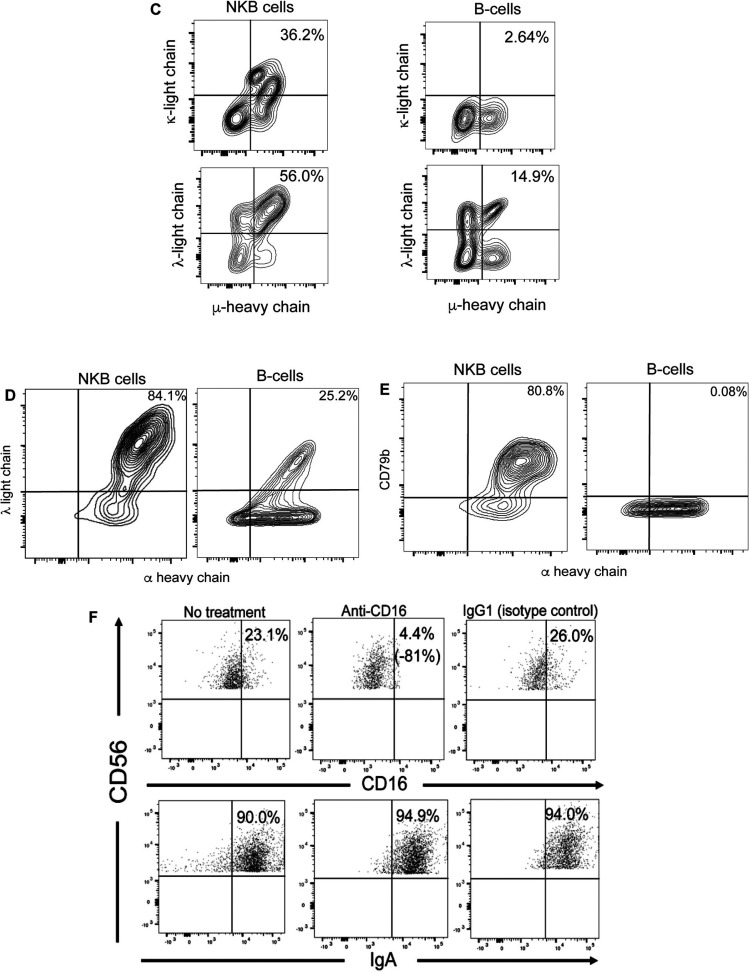
NKB cells in the SIV-infected colon possess immunoglobulin on their cell surface. (A) IgM, IgG, and IgA expression among NKB cells (top row) and B cells (bottom row) from 2 different SIV-infected colons. Symbols above each figure represent the symbols indicated in panel B. (B) Percentages of IgA, IgG, and IgM expressed by NKB cells and B cells among 9 different SIV-infected colons. Each symbol represents a different infected colon. (C) NKB and B cells stained for the surface λ or κ light chains and μ heavy chain. (D) NKB and B cells stained for the surface α heavy chain and λ light chain. (E) NKB and B cells stained for the surface α heavy chain and intracellular CD79b. (F) Cells from the colon were surface stained to identify NKB cells and then exposed to unlabeled anti-CD16 antibody or IgG1 (isotype control) for 30 min at 4°C. The cells were then stained with fluorochrome-conjugated anti-CD16 (same clone as the one for the unlabeled antibodies) or anti-IgA antibodies used to identify surface IgA.

Most B cells in the colon express IgA (30, 31). Indeed, we found that, on average, 83.8% (standard deviation [SD] = 9.6%) of B cells in the infected colon expressed the alpha heavy Ig chain (Fig. 3B). Therefore, we next determined the antibody (Ab) isotype on NKB cells in SIV-infected colons. An example of IgA, IgG, and IgM surface staining of NKB (top row) and B (bottom row) cells is shown in Fig. 3A. We found that, on average, 63.4% (SD = 30.3%) of NKB cells expressed the alpha heavy chain, 18.1% (SD = 29.0%) expressed the gamma heavy Ig chain, and 9.6% (SD = 9.5%) expressed the mu heavy chain (Fig. 3B). One animal’s colon had NKB cells that did not express an alpha heavy Ig chain (Fig. 3B). We also noted that IgM on NKB cells was more likely to possess λ light chains than κ light chains (Fig. 3C). This is also true of NKB cells expressing IgA (Fig. 3D). This finding suggests that the skewing of NKB cell light chains is more similar to that of mucosal plasma cells (32) than that of peripheral blood B cells, which have a greater predominance of κ light chains (33). Since surface Ig expression is dependent on Ig heavy chain interaction with CD79 (34, 35), we stained for CD79b expression. CD79b expression was associated with anti-IgA surface expression on NKB cells but not B cells (Fig. 3E). In contrast to CD79b, we observed similar expression levels of CD79a with NKB and B cells (Fig. 2D).

Previous reports in the literature have suggested that NKB cells are NK cells that nonspecifically bind antibodies used to stain the IgM on the cell surface via CD16 (36). To test this, we utilized a CD16-blocking antibody and found no difference in the ability of the anti-IgA antibody to bind to these cells, which is an IgG subclass antibody that would bind CD16, while the anti-CD16 antibody could not bind to NKB cells (Fig. 3F). These observations further suggest that the identification of the heavy Ig chain with antibodies is not due to the nonspecific binding of the antibody to CD16 on the NKB cell surface.

Our findings further indicate that NKB cells share phenotypic and functional characteristics with NK and B cells yet are different at specific levels (e.g., CD79b or class of heavy chain expressed) from NK cells and B cells, delineating them as unique cellular populations.

### NKB cells are a unique source of IL-18 in the colon after SIV infection.

**FIG 4:**
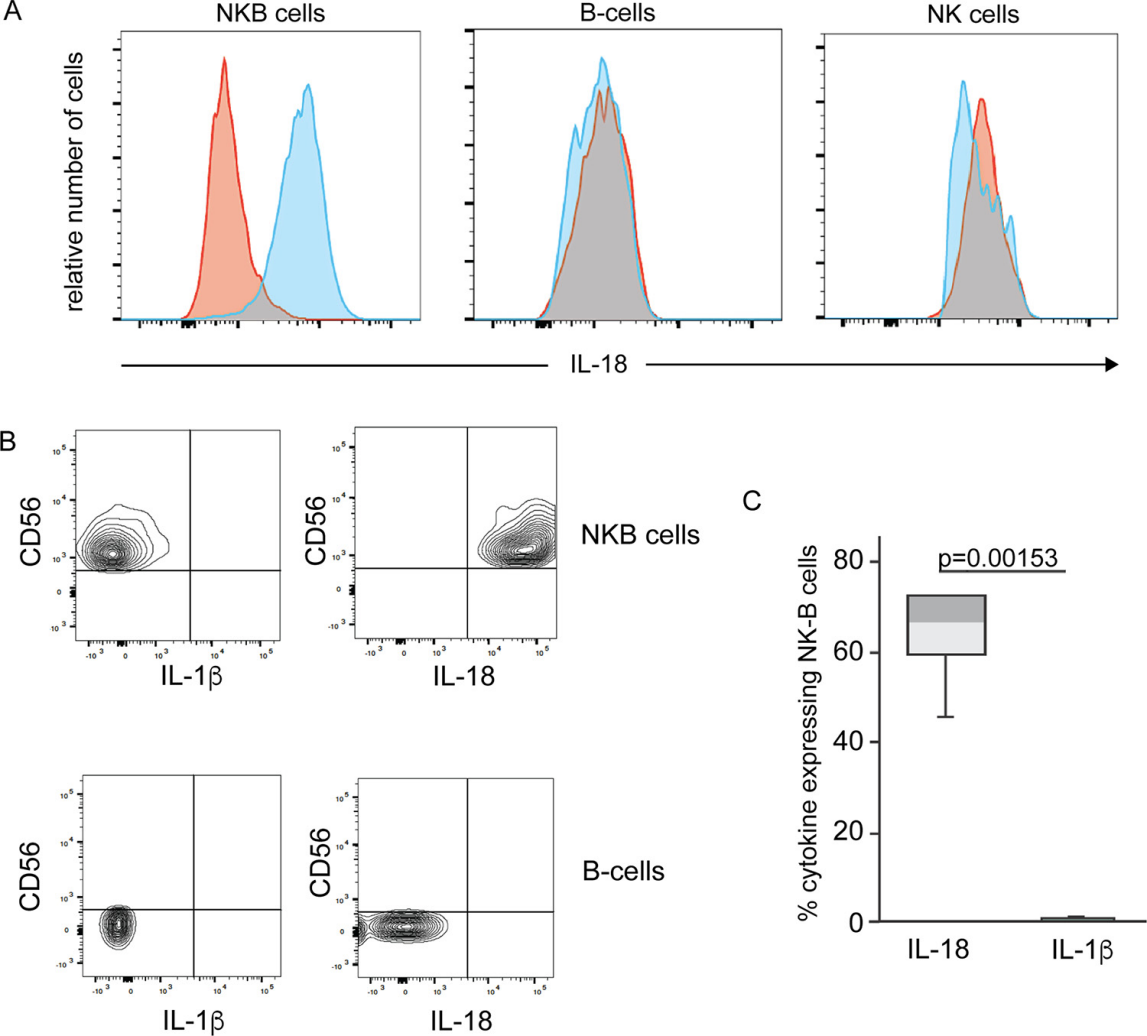
NKB cells but not NK cells or B cells in the SIV-infected colon express interleukin-18. (A) Histograms of CD45^+^ CD20^+^ CD56^+^ (NKB), CD45^+^ CD20^+^ CD56^−^ and CD45^+^ CD20^−^ CD56^+^ (NK) cells from an SIV-infected colon stained for interleukin-18 (IL-18) (blue histograms). Fluorescence-minus-one-stained cells were used as controls (red histograms). (B) Example of two-dimensional (2D) plots of CD56 versus IL-18 or IL-1β within NKB cells (top row) and B cells (bottom row). (C) Percentage of NKB cells expressing IL-18 and IL-1β within six SIV-infected colons. The horizontal lines in the boxes indicate the medians, boxes are the upper and lower quartiles, and vertical lines indicate variability outside the upper and lower quartiles. Individual points are outliers. The *P* values for statistical significance were determined using a Mann-Whitney U test. The *P* value threshold for significance was <0.05.

In a previous study with NKB cells in mice, the investigators noted that splenic NKB cells were capable of secreting IL-18 (28). Moreover, in response to GI pathogens, NKB cells were the sole source of IL-18 that triggered IFN-γ by innate lymphoid cell type 1 (ILC1) and NK cells (28). We found that dendritic cells, macrophages, and epithelial cells from the SIV-infected colon are not the primary source of IL-18 (data not shown). Therefore, we wanted to determine whether NKB cells in the GI tract were a source of IL-18. We stained lymphocytes from the lamina propria of SIV-infected colons with antibodies to IL-18 and markers for NKB cells. As controls, we also stained for IL-18 within NK cells and B cells. The cells were not stimulated *in vitro* since we wanted to determine whether IL-18 is naturally expressed by NKB cells. In six infected colon tissues, we found that 68% of NKB cells produced IL-18 (*P* = 0.002), whereas none of the B cells or NK cells contained IL-18 (Fig. 4). IL-18 and IL-1β are both canonically produced as a result of inflammasome activation (37); thus, we also stained for IL-1β and found that NKB cells produced little to no IL-1β (Fig. 4C), suggesting a noncanonical pathway of IL-18 production (37). Together, these findings suggest that NKB cells are a unique cellular source of the proinflammatory cytokine IL-18 but not IL-1β, which may lead to the downstream inflammation observed in the SIV-infected colon (28, 38).

### NKB cells possess a distinct transcriptome compared to NK cells and B cells.

To clarify the difference between NKB cells and NK and B cells in the colon, we isolated RNA from sorted NKB, NK, and B cells from the colons of three SIV-infected rhesus macaques and performed RNA sequencing (RNAseq). To verify that NKB cells were purified, we found that these cells expressed both MS4A1 (i.e., CD20) and NCR1 (i.e., NKp46), while B cells expressed only MS4A1, and NK cells expressed only NCR1 (see Table S1 in the supplemental material). MS4A1 and NCR1 transcript expression was confirmed by flow cytometry, in which we found CD20 and NKp46 expressed on NKB cells (Fig. 1 and 2). Below are some functionally important NKB cell transcripts expressed at relatively higher levels than in NK and B cells.

### Cytotoxic molecules in or on NKB cells.

**FIG 5:**
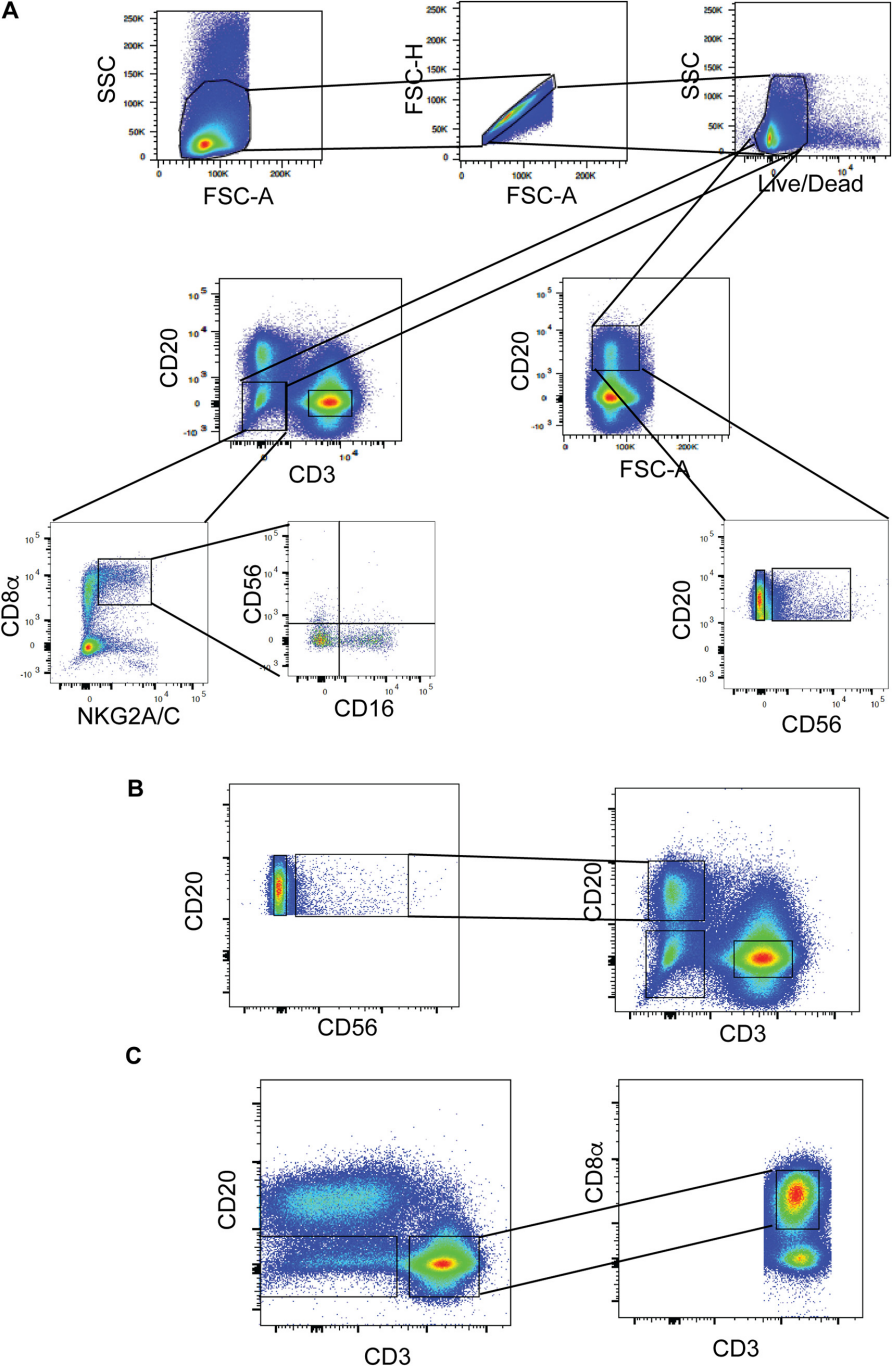

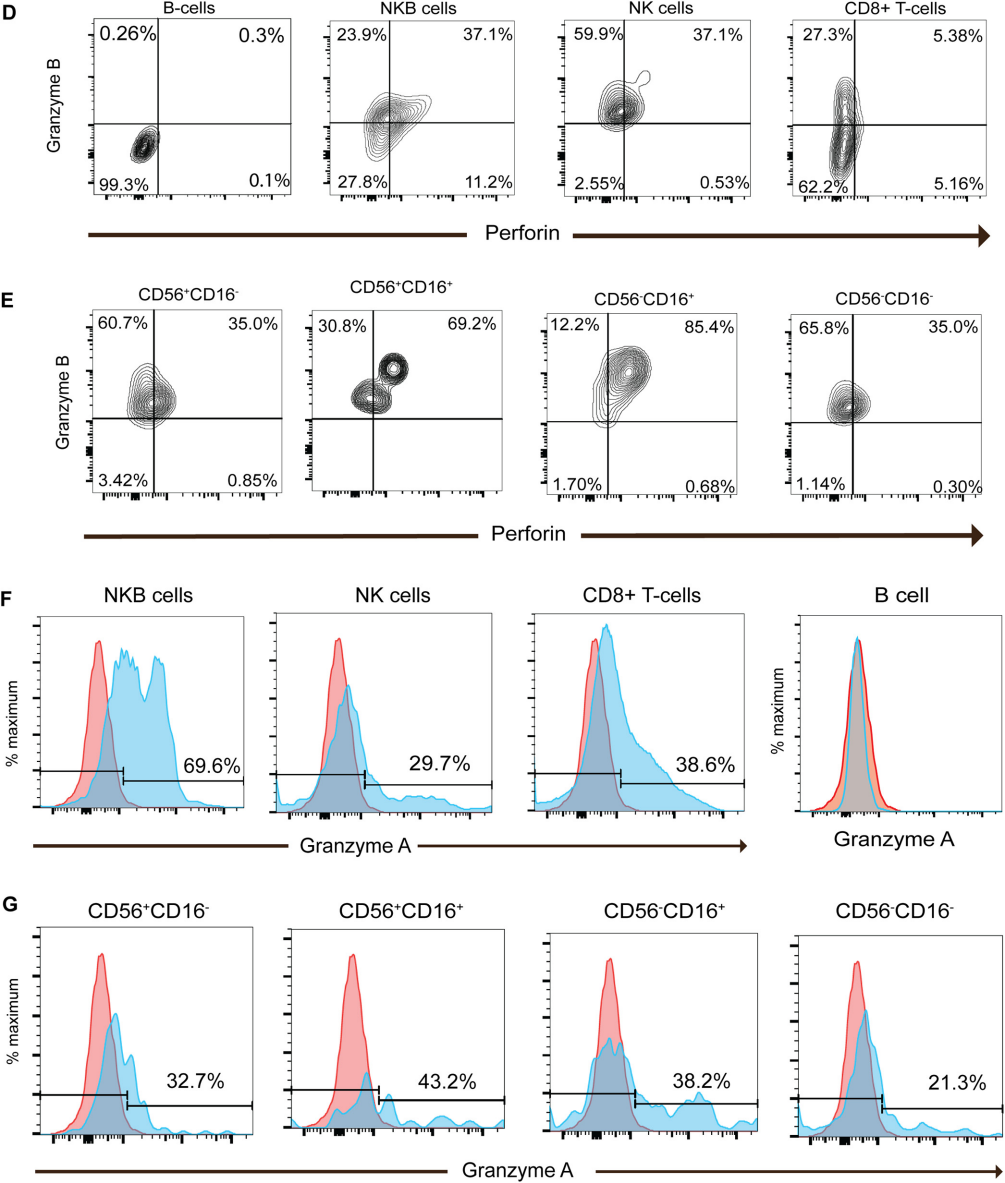

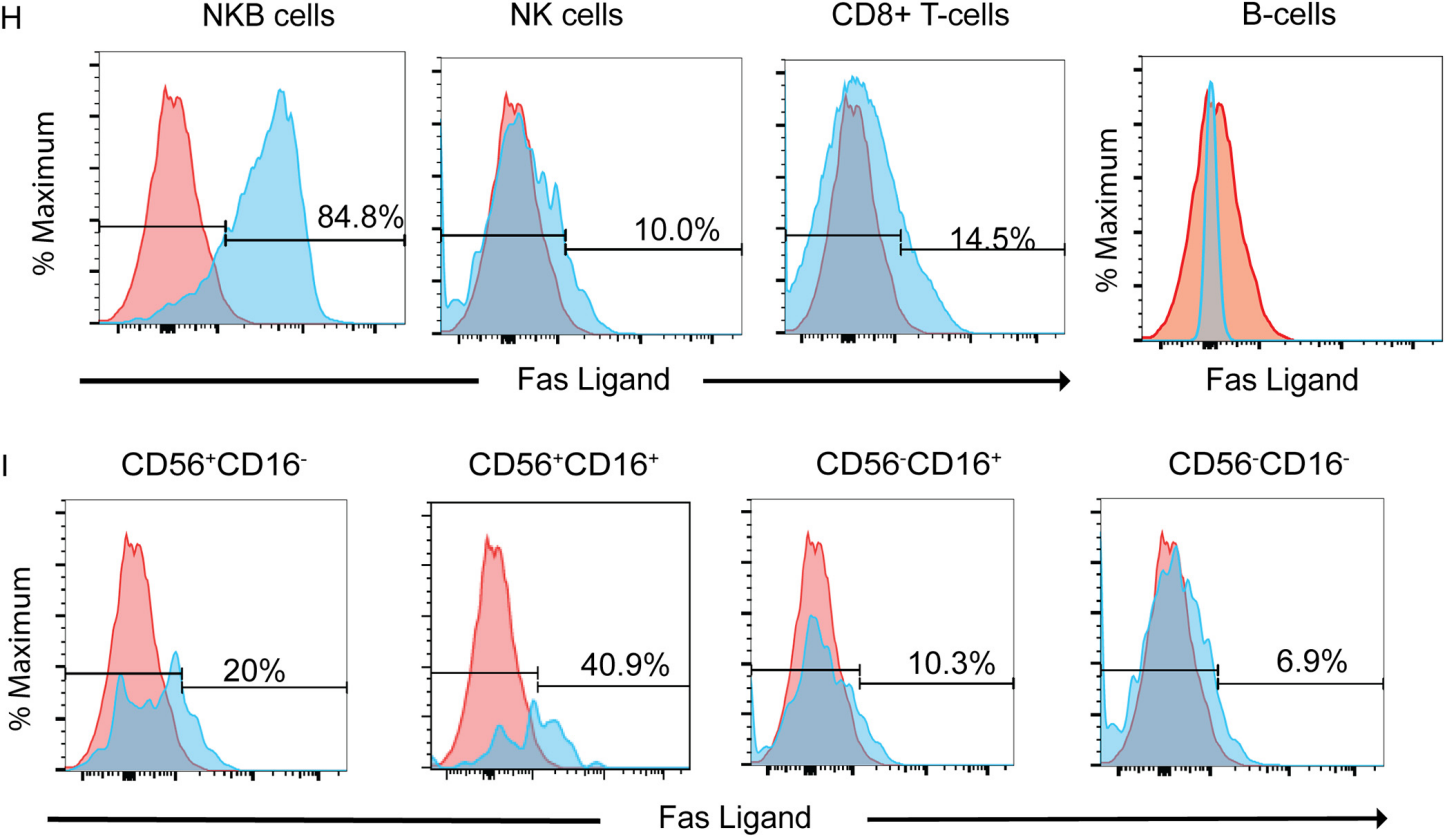
Frequency of NKB cells relative to NK cells and CD8^+^ T cells in the SIV-infected colon that express cytolytic molecules. (A) The approach used to identify NKB cell (CD45^+^ CD20^+^ CD56^+^) and NK cell (CD45^+^ CD202 CD3^−^ CD8a^+^ NKG2A/C^+^) subsets based on CD56 and CD16 staining by flow cytometry within the colons of SIV-infected macaques. (B) Gating strategy for NKB cells not expressing CD3. (C) Gating strategy used for identifying CD8^+^ T cells. (D) NKB cells, NK cells, and CD8^+^ T cells were evaluated by flow cytometry for the intracellular expression of perforin and granzyme B. Gates were set based on the intracellular expression of perforin and granzyme B within B cells. (E) Perforin and granzyme B coexpression within NK cell (NKG2A/C and CD8a CD3^−^ CD20^−^) subsets based on the differential expression of CD56 and CD16. Gates were set based on the intracellular expression of perforin and granzyme B within B cells. (F) NKB cells, NK cells, and CD8^+^ T cells were evaluated by flow cytometry for the intracellular expression of granzyme A (blue histograms). Gates were set based on granzyme A expression within B cells (red histograms), where expression was similar to that for the FMO control. (G) Granzyme A expression was measured within the various NK cell subsets (blue histogram). Gates were set based on granzyme A expression within B cells (red histograms). (H) NKB cells, NK cells, and CD8^+^ T cells were evaluated for Fas ligand (FasL) surface expression by flow cytometry (blue histograms). Gates were set based on FasL expression within B cells (red histograms), where expression was within the FMO control. (I) FasL expression on NK cell subsets based on the differential expression of CD56 and CD16 (blue histograms). Gates were set based on FasL expression on B cells (red histograms).

Regarding cytotoxic molecules, we found a 5-fold increase in granzyme H transcripts between NKB cells and NK cells (*P* = 0.023) and B cells (*P* = 0.0016). There was an 8-fold increase in granzyme A expression in NKB cells over B cells (*P* = 1.75 × 10^−21^) and an 11-fold increase over NK cells (*P* = 1.76 × 10^−5^). There was a 9-fold difference in Fas ligand (FasL) expression between NKB cells and NK cells (*P* = 0.0038) and B cells (*P* = 6.8 × 10^−5^). We stained leukocytes from the lamina propria of the infected colon with fluorochrome-conjugated antibodies directed to granzyme A and FasL to verify transcript expression. We could not stain for granzyme H due to the lack of anti-granzyme H Ab in macaques. As a control, we stained the cells intracellularly for granzyme B. Granzyme B’s transcript expression levels are similar between NKB cells and NK cells (Table S1). To identify NKB cells, we surface stained cells from SIV-infected colons with fluorochrome-conjugated anti-CD20 and -CD56. Although we initially broadly identified NK cells as CD45^+^ lineage-negative (Lin^−^) CD127^−^ CD56^+^ cells (38), in this confirmatory study, we further identified NK cells as NKG2A/C^+^ CD8α^+^ CD20^−^ CD3^−^ leukocytes, in line with more recent reports (39, 40). We then further broke down the NK cells (NKG2A/C^+^ CD8α^+^ CD20^−^ CD3^−^) based on the differential expression of CD56 and CD16 (Fig. 5A). We also show the presence of NKB cells even when we remove the CD3 cells (Fig. 5B). Figure 5C shows the gating used to define the CD8^+^ T cells in our study. Figure 5D shows a high level of staining for granzyme B in NK cells (NKG2A/C^+^ CD8α^+^ CD20^−^ CD3^−^), in particular the CD56^−^ CD16^+^ and CD56^+^ CD16^+^ subsets of NK cells (Fig. 5E), which agrees with previous studies using blood-derived NK cells (39, 40). Although NKB cells expressed granzyme B (Fig. 5D), the frequency of NKB cells expressing granzyme B (49.4 to 63.4%) was 1.7-fold lower than the frequency of granzyme B-positive NK cells (81.7 to 97.5%) but similar to that of CD8^+^ T cells (30.6 to 46.5%). Perforin was coexpressed with granzyme B in 37% of the NK cells and NKB cells, but very few CD8^+^ T cells expressed perforin (Fig. 5D). In contrast to granzyme B, many more NKB cells express granzyme A (47.9 to 69.6%) than NK cells (29.7 to 43.19%) or CD8^+^ T cells (18 to 38.6%) (Fig. 5F). Compared to NKB cells, few CD56^−^ CD16^+^ (38.2 to 40%) and CD56^+^ CD16^+^ (43.2 to 48.97%) NK cells express granzyme A (Fig. 5G).

Surface FasL triggers the destruction of virus-infected cells by NK cells (41). Our study noted a 9-fold increase of FasL transcripts in the NKB cell compared to the NK cell transcripts (*P* = 0.008). Therefore, we labeled surface leukocytes from the lamina propria of the infected colon for FasL. We found that 84.8% of NKB cells expressed surface FasL (Fig. 5H). In contrast, only 10% of total NK cells (20% present on CD56^+^ CD16^−^ NK cells) and 14.5% of CD8^+^ T cells expressed FasL. Even the highest frequency of FasL-expressing NK cell subsets (CD56^+^ CD16^+^) is less than half the frequency of FasL^+^ NKB cells (Fig. 5I).

### NKG2D activation receptors on NKB cells.

**FIG 6:**
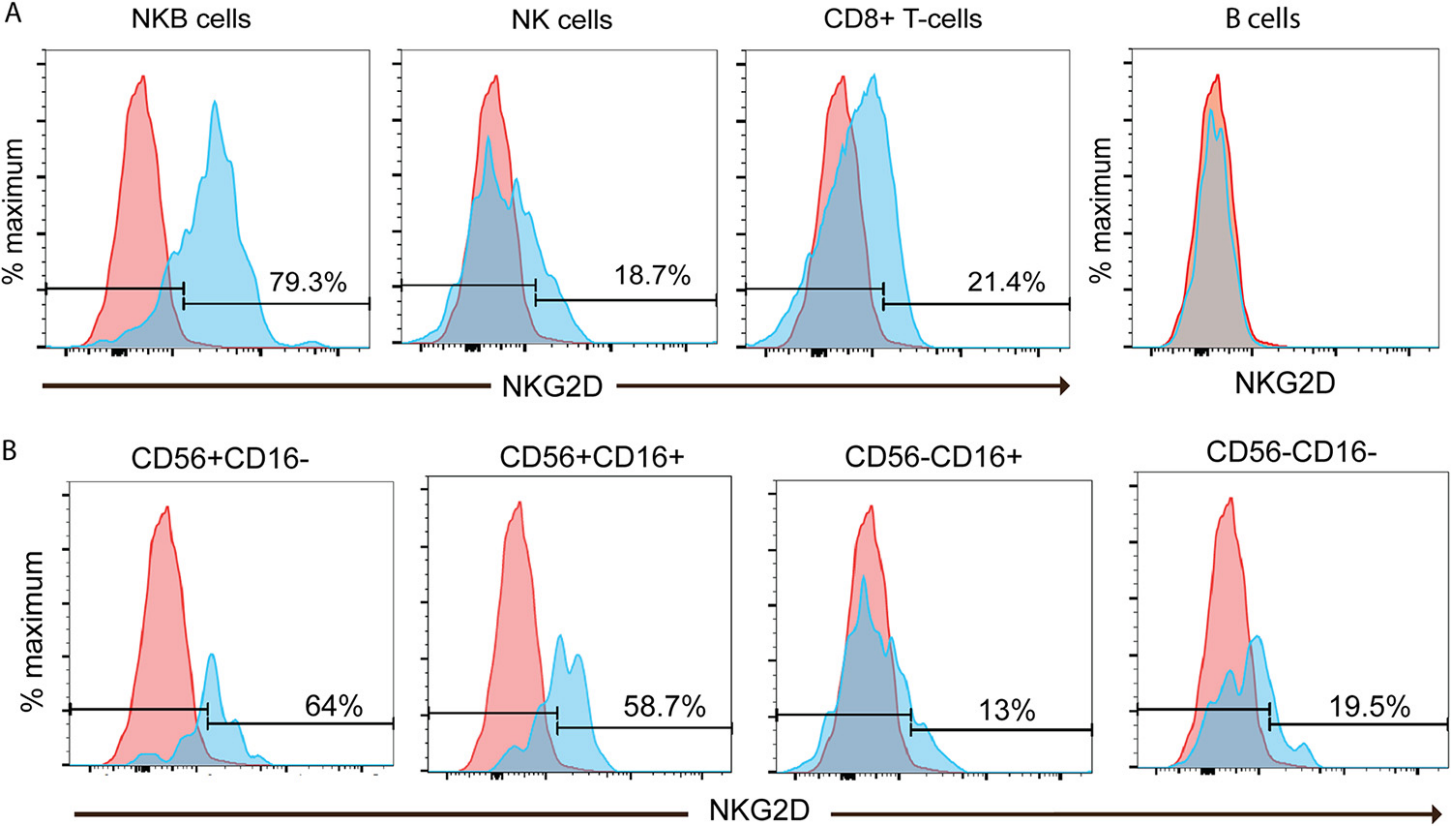
Frequency of NKB cells from SIV-infected colons expressing surface NKG2D. (A) NKB cells, NK cells, and CD8^+^ T cells were evaluated for the surface expression of NKG2D by flow cytometry (blue histograms). Gates were set based on NKG2D expression within B cells (red histograms), where expression was within the FMO control. (B) NKG2D expressed on NK cell (NKG2A/C^+^ CD8α CD3^−^ CD20^−^) subsets from SIV-infected colons based on the differential expression of CD56 and CD16 (blue histograms). Gates were set based on the surface expression of NKG2D on B cells (red histograms).

In addition to lytic molecules, we also asked if activation receptors critical for triggering the lysis of infected cells by NK cells are also present on NKB cells. We and others had shown that NK cells from the blood lysed autologous HIV-infected cells when NKG2D was engaged (42, 43). Although there is a 4-fold increase in transcripts for NKG2D in NKB cells relative to NK cells and B cells, the difference was not statistically significant. Nevertheless, we found that the frequencies in the SIV-infected colon of NKG2D^+^ NK cells (18.7 to 60.4%) and CD8^+^ T cells (21.4 to 41.2%) were similar to or lower than those of NKG2D^+^ NKB cells (70.8 to 79.3%) (Fig. 6A). However, when further examining the CD56^−^ CD16^+^ subset of NK cells, which is the most cytotoxic subset of NK cells (37, 38), on average, there were ∼20% fewer NKG2D^+^ NK cells (58.7%) than NKG2D^+^ NKB cells (Fig. 6A and B).

### In the infected colon, NKB cells express higher transcript and protein levels of IFN-γ than traditional NK cells.

**FIG 7:**
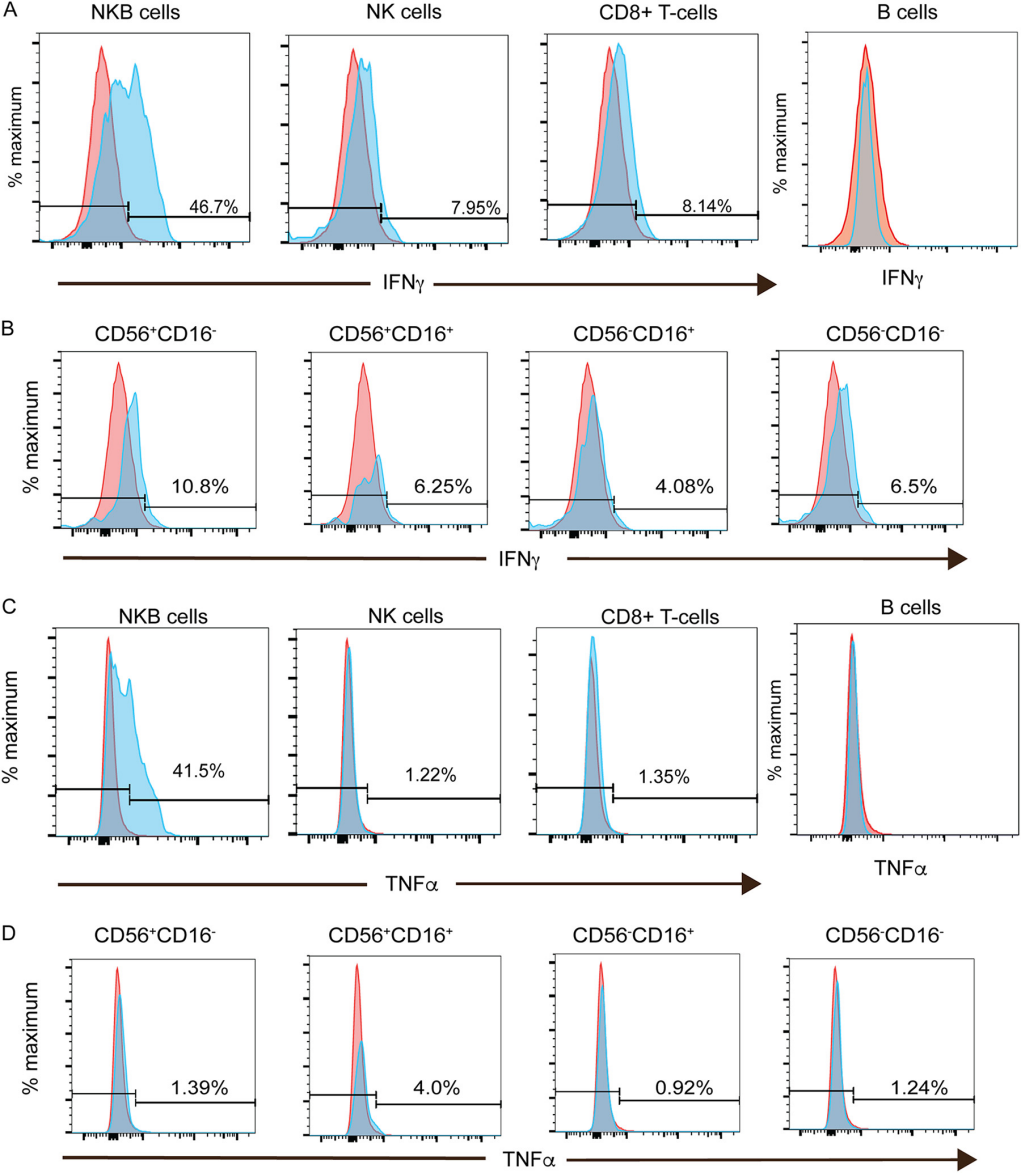
Frequency of NKB cells relative to NK cells and CD8^+^ T cells in the SIV-infected colon expressing interferon gamma and tumor necrosis factor alpha. (A and C) NKB cells, NK cells, and CD8^+^ T cells were evaluated by flow cytometry for the intracellular expression of IFN-γ (A) and TNF-α (C) (blue histograms). Gates were set based on the intracellular expression of IFN-γ and TNF-α within B cells (red histograms), where expression was within the FMO controls. (B and D) IFN-γ (B) and TNF-α (D) within NK cell (NKG2A/C^+^ CD8α^+^ CD3^−^ CD20^−^) subsets based on the differential expression of CD56 and CD16 (blue histogram). Gates were set based on NKG2D expression on B cells (red histograms).

It has been previously described (28) that one of the primary functions of NKB cells is to secrete proinflammatory cytokines (i.e., IL-18) to stimulate ILC1s and NK cells to secrete inflammatory cytokines such as interferon gamma (IFN-γ). Thus, we examined the expression of cytokine transcripts in our RNAseq data set. There was an 11-fold increase in the expression of transcripts for IFN-γ in NKB cells compared to NK cells (*P* = 0.004) and B cells (*P* = 2.3 × 10^−5^) (Table S1). We then confirmed the frequency of NKB cells expressing IFN-γ by utilizing flow cytometry. The frequency of IFN-γ-expressing NKB cells was higher (46.7%) than the frequency of either NK cells (7.95%) or CD8^+^ T cells (8.14%) expressing IFN-γ (Fig. 7A). This was also true for the NK cell subsets (Fig. 7B). While we did not note changes to TNF-α transcripts between NKB cells and NK cells or B cells (Table S1), we observed that 41.5% of NKB cells expressed TNF-α, even though few if any NK cells (including NK cell subsets) or CD8^+^ T cells from the same colon expressed TNF-α (Fig. 7C and D).

**FIG 8:**
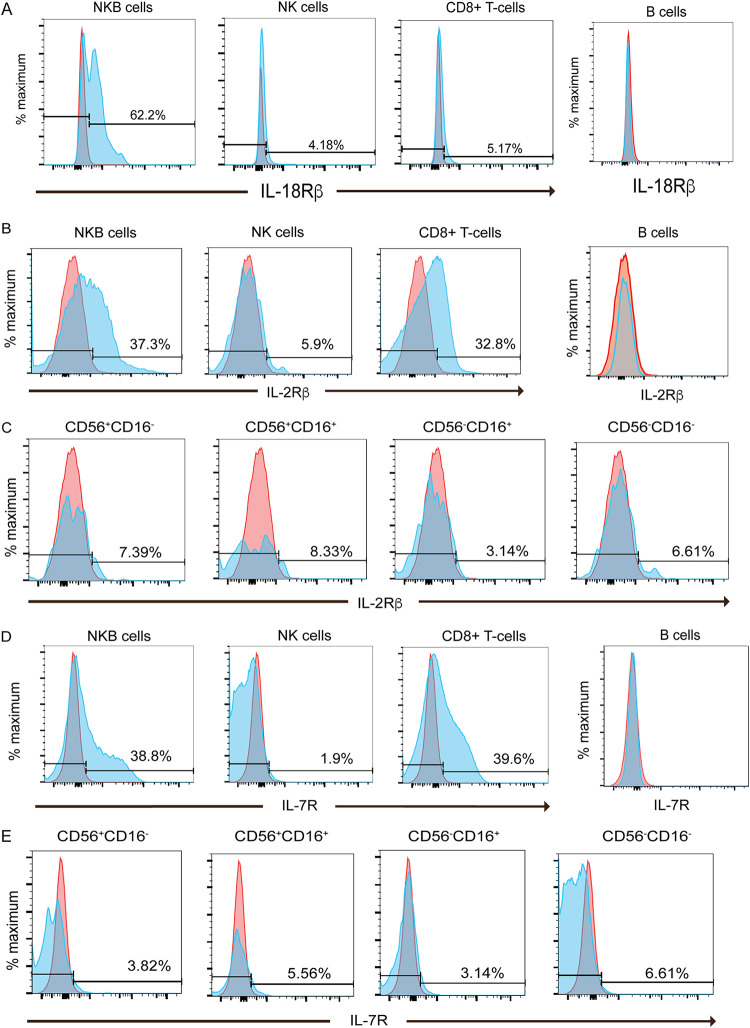
Frequency of NKB cells relative to NK cells and CD8^+^ T cells in the SIV-infected colon expressing surface interleukin-18 receptor beta, interleukin-2 receptor beta, and interleukin-7 receptor. (A) NKB cells, NK cells, and CD8^+^ T cells were evaluated by flow cytometry for the surface expression of IL-18Rβ (blue histograms). Gates were set based on the expression of IL-18Rβ within and on B cells (red histograms). (B and D) NKB cells, NK cells, and CD8^+^ T cells were evaluated by flow cytometry for the surface expression of IL-2Rβ (B) and IL-7R (D) (blue histograms). Gates were set based on the intracellular expression of IL-2Rβ and IL-7R within B cells (red histograms). (C and E) IL-2Rβ (C) and IL-7R (E) on NK cell (NKG2A/C^+^ CD8α^+^ CD3^−^ CD20^−^) subsets based on the differential expression of CD56 and CD16 (blue histograms). Gates were set based on the intracellular expression of IL-2Rβ and IL-7R within B cells (red histograms).

Given that engaging the IL-18 receptor (IL-18R) increases IFN-γ/TNF-α expression in lymphocytes (44), we wanted to determine the extent of IL-18 receptor expression on NKB cells. We found an 11-fold increase in the expression of IL-18Rβ transcripts in NKB cells relative to the same transcripts found in NK cells (*P* = 0.0034) and B cells (*P* = 1.28 × 10^−5^) (Table S1). By flow cytometry, we found that a higher percentage of NKB cells (62.2%) expressed the IL-18Rβ chain on their surface than NK cells (4.18%) or CD8^+^ T cells (5.17%) (Fig. 8A). We noted that IL-18 was expressed by NKB cells but not NK cells (Fig. 4) or CD8^+^ T cells (data not shown).

### NKB cells have a greater propensity to proliferate than NK cells in the SIV-infected colon.

We also noted increases in transcripts from NKB cells for the receptors of two cytokines critical for the proliferation and survival of NK cells, IL-2Rβ and IL-7R (45–47). Here, we found 4-fold and 8-fold increases in transcripts for IL-2Rβ relative to those in B cells (*P* = 2.99 × 10^−5^) and NK cells (*P* = 0.0067), respectively. IL-7R transcripts were increased in NKB cells by 10-fold compared to those in B cells (*P* = 0.0001) and NK cells (*P* = 0.0051). The expression of the cytokine receptor transcripts was then confirmed by flow cytometry, where we found very few, if any, NK cells expressing either IL-2Rβ (Fig. 8B and C) or IL-7R (Fig. 8D and E). In contrast, 37.3% of NKB cells express IL-2Rβ, and 38.8% of NKB cells express IL-7R. As a positive control, we stained CD8^+^ T cells and found comparable expression levels.

**FIG 9:**
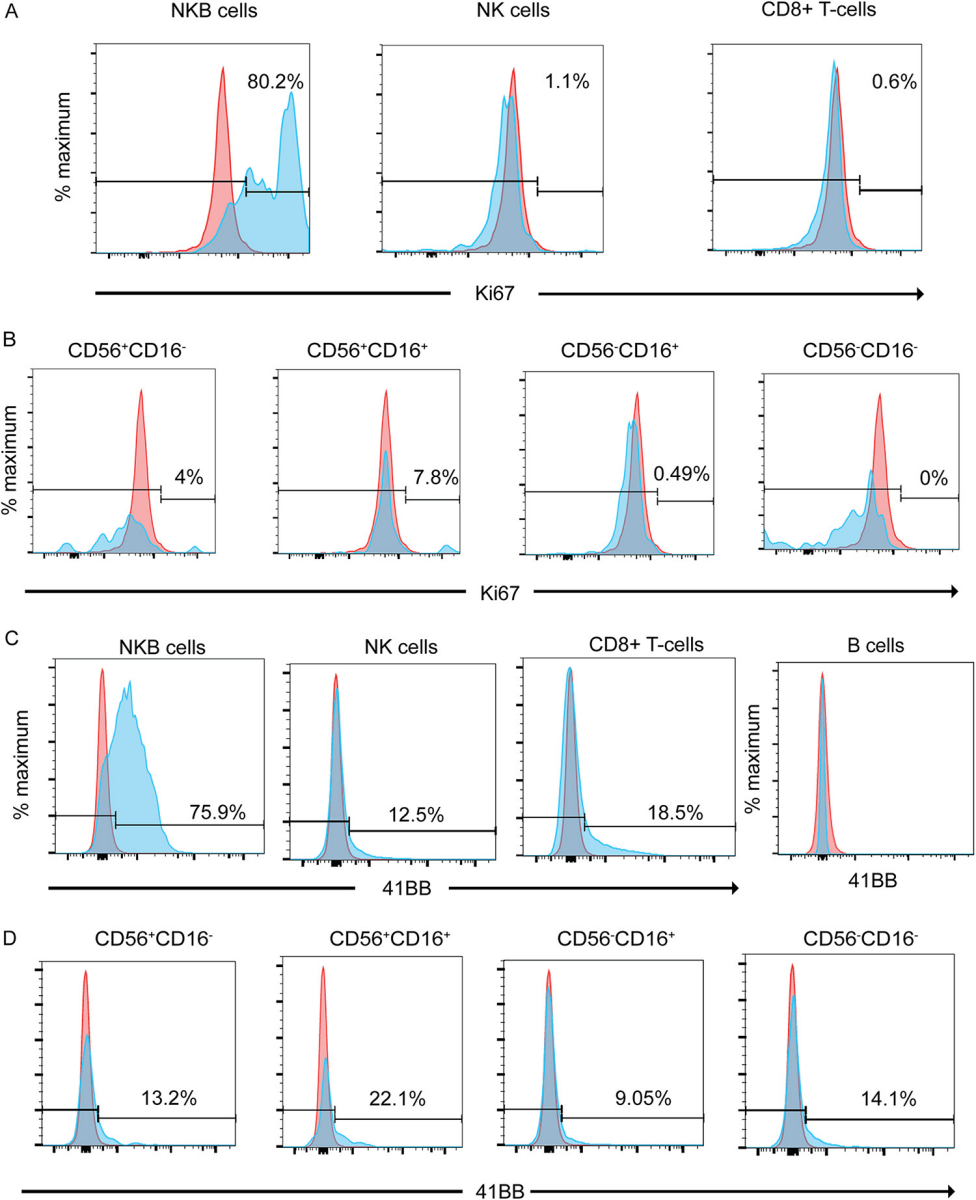
41BB surface expression and proliferation of NKB cells relative to NK cells and CD8^+^ T cells in the SIV-infected colon. (A) NKB cells, NK cells, and CD8^+^ T cells within SIV-infected colons were evaluated for proliferation by flow cytometry by detecting the marker Ki67 (blue histograms). (B) Ki67 was also assessed in NK cell (NKG2A/C^+^ CD8α^+^ CD3^−^ CD20^−^) subsets based on the differential expression of CD56 and CD16 (blue histograms). (C) Flow cytometry was used to evaluate the surface expression of 41BB on NKB cells, NK cells, and CD8^+^ T cells within the SIV-infected colon (blue histograms). (D) Surface expression of 41BB on NK cell subsets in the SIV-infected colon (blue histograms). Gates were set based on the expression of Ki67 and 41BB within and on B cells (red histograms).

Since IL-7R and IL-2Rβ are both utilized as receptors for cytokines that trigger the proliferation of NK cells, we asked whether NKB cells also could proliferate. Our RNAseq data showed that MKi67 transcripts for NKB cells (*P* = 0.03) were 8-fold higher than those for NK cells. To confirm the proliferation potential, we then utilized intranuclear staining for Ki67. We found that 80.2% of NKB cells express Ki67, while only 1.1% of NK cells and 0.6% of CD8^+^ T cells express Ki67 (Fig. 9A), suggesting the high proliferative potential of NKB cells. Relatively low levels of Ki67 expression (<7.8%) were found in all four NK cell subsets (Fig. 9B) compared to Ki67 expression by NKB cells.

In addition to cytokine-stimulated proliferation, triggering 41BB on NK cells can lead to increased proliferation (48); thus, we wished to examine the expression of 41BB on NKB cells. We observed a 5-fold increase in transcripts for 41BB expression on NKB cells compared to NK cells (*P* = 0.0325). Further confirming this via flow cytometry, we found that 75.9% of NKB cells express surface 41BB, while only 12.5% of the NK cell population and 18.5% of the CD8^+^ T-cell population did (Fig. 9C). In addition, when looking carefully at the NK cell subset, at best, 22.1% of CD56^+^ CD16^+^ NK cells expressed 41BB (Fig. 9D).

## DISCUSSION

We found that (i) NKB cells are present in the lamina propria of the SIV-infected colon but are present in limited numbers in the uninfected colon; (ii) NKB cells share receptors, markers, and functions with NK cells and B cells; and (iii) NKB cells are a source of IL-18, IFN-γ, and TNF-α in the SIV-infected colon. We also noted that NKB cells possess a distinct transcriptome compared to NK cells and B cells and, therefore, appear to be a unique cell population. (iv) NKB cells are a highly proliferative cell population in the SIV-infected colon relative to NK cells and CD8^+^ T cells.

Our findings are in contrast to those of a subsequent small study (36) of the initial study of NKB cells in mice (28). In this subsequent study of NKB cells, the investigators reported that CD19^+^ cells do not express the NK cell activation receptor NKp46. Therefore, NKB cells are not a population distinct from B cells or NK cells. They report that NKB cells instead display conventional B cells’ phenotypic and functional characteristics. Like this small study, we used a stringent gating strategy for cells within the lamina propria of SIV-infected and uninfected colons, which was designed to preserve rare and actual events designated NKB cells while excluding potential artifacts generated by doublets, dead cells, antibody aggregates, or autofluorescent cells. While there may be characteristics that NKB cells share with NK cells and B cells, we found many features based on RNAseq, functional assays, and flow cytometry that reveal a unique cell type.

A caveat to our study is that initially, we defined NK cells as lineage-negative (Lin^−^) CD56^+^ CD127^−^ cells. ILCs (Lin^−^ CD127^+^) also express CD56, so to rule them out, we evaluated only NK cells that were CD56^+^ CD127^−^. Our study noted that none of the NK cell subsets appear to express CD127 (Fig. 7). However, by selecting for CD56^+^ NK cells, we may have overlooked a subpopulation of NK cells defined in macaques as NKG2C/NKG2A^+^ CD8α^+^ CD20^−^ CD3^−^ lymphocytes that lack CD56 (39, 40). Most NK cells with cytotoxic potential in macaque monkeys based on granzyme B and perforin expression are CD56^−^ CD16^+^ (40) (Fig. 5). In our later flow studies (Fig. 5 to 9), we confirmed that in our lineage-negative CD56^+^ CD127^−^ RNAseq data, almost all of the differences described in the RNAseq data could be related to NKG2C/NKG2A^+^ CD8α^+^ CD20^−^ CD3^−^ NK cells, which appear to lack CD127 (Fig. 8).

Our data illustrate that besides the lamina propria of the infected colon, draining mesocolic LNs also contain NKB cells (Fig. 1D). A more recent study also shows NKB cells in uninfected rhesus macaques and human blood, lymph nodes, and even GI tissues (29). The difference in the outcomes of our studies may be due to how we selected NKB cells. Whereas the other investigator selected for CD20^+^ NKG2A/C^+^ CD127^−^ cells, we chose to look at CD20^+^ CD56^+^ cells without selecting for CD127. We found that not all NKB cells express NKG2A/C (Fig. 2C), and therefore, we decided not to use this as a marker to identify NKB cells. Moreover, NKB cells also expressed IL-7 receptors (Fig. 8), and consequently, we included this subpopulation of cells in our analysis.

Our study demonstrates that NKB cells are present in SIV-infected colons and nearly undetectable in uninfected colons (Fig. 1A). This is the case regardless of whether they are rhesus or cynomolgus macaques (Fig. 5A). Like rhesus macaques, cynomolgus macaques lose CD4^+^ T cells, contain microbial translocation, and develop inflammation when the gut is infected with SIVmac239 (49). Although we did not find any difference whether we used rhesus (Fig. 1A) or cynomolgus (Fig. 5A) monkeys in evaluating for NKB cells, it would have been desirable to use the same species of SIV-infected monkey throughout our study. We used only cynomolgus monkeys in the studies shown in Fig. 5 to 9 because (i) these were the only discarded colons that we could obtain at the time when this part of the study was initiated and (ii) we did not see any difference in the data obtained in Fig. 1 and 5.

Another caveat to our data is that comparing NKB cells’ presence in uninfected, acutely infected, and chronically infected colons was a cross-sectional study rather than a study involving lymphocytes from the same animal over time. Although a time course study would be ideal, samples in this study were provided to us as “discarded tissue” after the completion of experiments by other investigators at the primate center. We will endeavor to perform time course studies in the future. Even more desirable, for clinical relevance, is to see whether colon samples from humans infected with HIV but not uninfected humans contain NKB cells.

Our studies show that NKB cells are a distinct population of cells that have the properties of NK cells and B cells. Besides the coexpression of CD56 and CD20 (Fig. 1 and 5), NKB cells express Ig molecules on their surface (Fig. 3), particularly IgA, predominately with λ over κ light chain expression. In contrast, in normal B cells in circulation, the κ light Ig chain is more than twice as likely to be found as the λ light Ig chain (33). Typically, rearranged VJ genes associate with κ alleles before moving to λ alleles during allelic exclusion (33). However, in the GI tract, the λ light Ig chain appears to dominate over the κ light Ig chain, primarily associated with the α heavy Ig chain (32). In the GI tract, the *Ig*λ locus has “nested” V and J segments that can recombine to generate new rearrangements on the same allele, and there is no known mechanism for the inactivation of the *Ig*λ locus, unlike the *Ig*κ locus, which is typically done by κ-deleting element (50). The intact *Ig*λ locus is also available for secondary rearrangements following *Ig*λ expression. Thus, NKB cells in SIV-infected colons have IgA, similar to what is observed with B cells in the gut.

IgA expression is prevalent on B cells in the GI tract (30, 31). Many of these B cells become plasma cells capable of secreting dimeric IgA with the J chain. We found that NKB cells express the J chain. We also noted that the J chain in NKB cells is absent in CD8^+^ T cells (data not shown). If NKB cells in the SIV-infected colon can differentiate into plasma cells, they would be capable of secreting dimeric IgA. We also noted the coexpression of CD79b and IgA (Fig. 3E). This finding is of great interest since CD79 is a signal-transducing molecule, and thus, Ig could potentially act as an antigen-specific trigger for NKB cells (34, 51). Moreover, the association of CD79b with the α heavy Ig chain (Fig. 3E) essentially rules out any role for a poly-IgA receptor on NKB cells leading to monomeric IgA surface expression (34, 35). Although CD79a is also expressed, staining was limited and likely a consequence of using a monoclonal antibody to a human epitope that may not be optimal for macaque antigen (Fig. 2D). It remains to be seen what the antigens that bind to IgA on NKB cells in the infected colon are.

Although CD79b is expressed with α heavy Ig chains within NKB cells, this appears not to be the case for B cells (Fig. 3E). This may not be surprising given that affinity maturation occurs in the germinal centers of secondary follicles of lymphoid tissues. Downmodulation of CD79b is essential for B-cell selection in the germinal center (52) and possibly antigen presentation to T follicular helper cells (53). This may not be the case for NKB cells. Although NKB cells express markers associated with B cells found in the germinal center (e.g., CCR5), it is plausible that NKB cells are unable to undergo selection in the germinal centers or are not present in these sites. This will need to be verified in future studies by histology and the use of B-cell markers such as CD38 and GL7 to indicate the stage of differentiation to determine if CD79b downmodulation occurs at all in NKB cells.

Previous studies in mice (28) indicate that NKB cells may have originated from a common B-cell precursor (i.e., pro-B cells). However, our data point to the possibility that NKB cells in the colon may have been derived from NK cells. It is believed that Ig loci within NK cells do not undergo V(D)J recombination, and NK cells are present at average numbers in RAG-deficient mice (54–56). However, studies using RAG fate-mapped mice have shown that ∼40% of NK cells are derived from RAG-expressing common lymphoid progenitors (57). In addition, many of the RAG-expressing NK cells were long-lived memory cells (57). Interestingly, we noted that NK cells express the μ heavy Ig chain, λ light Ig chain, and κ light Ig chain in the cytoplasm but not on the cell surface (data not shown). We also observed that <10% of CD56^−^ CD16^+^ NK cells express intracellular IgM, while >40% of other NK cell subsets in the SIV-infected colon express intracellular IgM. It is possible that the CD56^−^ CD16^+^ NK cell subsets could be effector cells only and do not become NKB cells, while NKB cells develop from RAG-expressing CD56^+^ NK cells. We speculate that other B-cell molecules (e.g., CD20) begin to appear upon SIV infection, including CD79, which are critical for Ig surface expression (34). We support this by finding that CD79b is not found within NK cells from SIV-infected colons, even though intracellular IgM is present there (data not shown). Unlike IgA-bearing B cells with downmodulated CD79b, IgM-bearing B cells express CD79b (52). Thus, more studies are needed to determine the mechanism leading to Ig expression in NK and NKB cells in the infected colon to determine whether NKB cells arise from pro-B or NK cells.

NKB cells are cytotoxic and secrete inflammatory cytokines such as IFN-γ and TNF-α (Fig. 8), similar to the functional properties of NK cells. Not only do NKB cells possess granzyme B and perforin-like NK cells in the GI tract (Fig. 5D), but a large percentage of them also express granzymes A and K (Fig. 2A and Fig. 5F). In fact, in both the RNAseq and flow cytometry data, a higher frequency of NKB cells expressed granzyme A than NK cells or CD8^+^ T cells (Fig. 5F). Furthermore, there were two subpopulations of granzyme A-expressing NKB cells (Fig. 5F). The significance and phenotypes of these subpopulations remain of interest to us and need to be further examined. In recent studies, peripheral blood CD8^+^ T cells possessing granzyme B lysed macrophages infected with HIV rather than CD4^+^ T cells infected with the same virus (58). CD8^+^ T cells possessing granzymes A, K, and/or M were likely to lyse HIV-infected CD4^+^ T cells (58). Moreover, granzyme A cleaves gasdermin B (59) to trigger nonconical pyroptosis (59). Gasdermin B expression is increased by IFN-γ binding to its receptors (59). Other investigators have shown that pyroptosis mechanisms may account for the loss of CD4^+^ T cells during HIV infection (14, 60, 61). We will therefore determine whether granzyme A may account for the higher levels of CD4 loss within the GI tract by inducing pyroptosis.

IFN-γ and TNF-α are enhanced within NKB cells, but what triggers these in the cells may be elusive. IL-18 binding to its receptor leads to the increased and stable expression of IFN-γ and TNF-α in NK cells (44, 62). In our RNAseq and flow cytometry data (Fig. 8A), higher frequencies of NKB cells express IL-18Rβ. Given the increased expression of IL-18 (Fig. 4) found in our study, we reason that IL-18 produced by NKB cells binds to its receptor on NKB cells, leading to the increased expression of IFN-γ and TNF-α. This is increased by the engagement of CD94/NKG2C by HLA-E (62). It is plausible that IFN-γ could account for the loss of CD4^+^ T cells without any impact on CD8^+^ T cells (63), and TNF-α could increase microbial translocation (21, 22, 24, 64). TNF-α has been known to trigger the loss of tight junction formed between epithelial cells by the downmodulation of tight junction proteins (24, 65). The mechanisms by which CD4^+^ T cells are lost through IFN-γ could be numerous, although it has been speculated to involve increases in PD1 and Fas expression by CD4^+^ T cells (63).

Our study also demonstrates that most NKB cells express FasL, while only low frequencies of NK cells or CD8^+^ T cells do so (Fig. 5H). FasL has been known to trigger Fas on CD4^+^ T cells during HIV infection (66). Moreover, the triggering of Fas leads to a drastic loss of CD4^+^ T cells in the GI tract during the acute phase of SIV infection (13). Since a loss of CD4^+^ T cells, mainly T_H_17 cells (9, 67), is seen during infection, especially in the GI tract, it may be possible that the appearance of NKB cells could lead to a loss of CD4^+^ T cells. T_H_17 cells are sensitive to Fas-mediated destruction (15), and therefore, further studies are necessary to determine if their preferential loss may be due in part to FasL on NKB cells more so than NK cells or CD8^+^ T cells.

The proliferation of NK cells is impacted by IL-7, IL-15, and 41BB (47, 48, 68). We noted that NKB cells have higher levels of transcripts leading to IL-7R, IL-2Rβ (which is also the beta chain receptor for IL-15), and 41BB but also to MKi67 than do NK cells. These data were confirmed by flow cytometry (Fig. 8 and 9). Ki67 is a protein that acts as a marker of proliferation and is essential for the cell to transition out of the G_1_ phase of the cell cycle (69). Although CD8^+^ T cells in the infected colon also stain for IL-7R and IL-2Rβ (Fig. 8), very few CD8^+^ T cells express 41BB and Ki67 (Fig. 9). Few NK cells express IL-7R, IL-2Rβ, 41BB, and Ki67 proteins as detected by flow cytometry (Fig. 8 and 9). The distinct contribution of each cytokine and receptor-ligand interaction to the proliferative potential of NKB cells merits further study.

## MATERIALS AND METHODS

### Ethics statement.

The tissues were provided by the Nonhuman Primate Biological Materials Distribution Core of the Wisconsin National Primate Research Center (University of Wisconsin at Madison). Tissues from this core have been harvested ante- or postmortem from animals assigned to the University of Wisconsin at Madison IACUC-approved research protocols. Because we acquired discarded postmortem tissue, specific protocol numbers are not provided.

### Animals and virus.

This study used 10 Indian-origin rhesus macaques (Macaca mulatta) and 11 cynomolgus macaques (Macaca fascicularis). The monkeys (12 males and 9 females; average age, 5.8 years [range, 1.5 to 12 years]) were infected with SIVmac239. Uninfected animals in our study were age and gender matched with infected animals. Animals infected with SIV who had no overt clinical disease signs were sacrificed using humane approaches between 1 and 24 months following infection. Mesocolic lymph nodes and colons were necropsied immediately, and tissue was placed on ice until being picked up from the facility and processed in a biosafety level III facility at the AIDS Vaccine Research Laboratory at the University of Wisconsin (<30 min after necropsy).

### Cell isolation and processing.

Colon tissue sections (average area, 6 cm by 9 cm) were processed immediately for single-cell suspensions. Fatty tissue and feces were removed, and tissue was cleaned and washed in phosphate-buffered saline (PBS) before weighing. For colons, the mean weight ± standard deviation was 14.98 ± 4.8 g. Colon sections were first manually dissected into 2.0-cm sections and then treated with 0.33 M dithiothreitol (DTT; Sigma, St. Louis, MO) in Hanks balanced salt solution (HBSS) and Primocin (Fisher Scientific, Pittsburgh, PA) to remove the mucus. Next, 5-mm strips of tissues were further incubated for two 50-min cycles (37°C with constant shaking at 225 rpm) with 2 mM EDTA (Gibco) and 0.1% bovine serum albumin (BSA; Fisher) in HBSS to remove intraepithelial leukocytes. Finally, tissue was minced into 1-mm pieces and treated for two 60-min incubations (37°C with shaking at 225 rpm) with 10 mg/mL of collagenase II from Clostridium histolyticum (Gibco) in RPMI 1640 medium containing 2% BSA and Primocin to isolate lamina propria leukocytes. The mesocolic lymph nodes were minced into 1-cm sections and digested with collagenase as described above for colon tissue. The mesocolic lymph nodes weighed an average of 7.5 ± 1.1 g. In all steps, cell suspensions were filtered through 100-μm mesh. After counting, we moved the cells into recovery cell culture freezing medium (Gibco). We froze the contents in cryovials (Corning) at a rate of 1°C per min in a −80°C freezer overnight hours before transfer to liquid nitrogen for cryopreservation.

### Flow cytometry.

Flow cytometry was conducted as previously described (38). In brief, cryovials containing frozen cell suspensions were immediately thawed following removal from liquid nitrogen, washed twice in PBS (Gibco), and counted. No Golgi-blocking additives were used (e.g., monensin or brefeldin). At least 10E6 cells were stained in test tubes (Fisher). Cells were first stained with a 1:1,000 dilution of Aquadead staining dye (Invitrogen) for 20 min at room temperature in PBS. Cells were then washed with PBS twice to remove the residual dye. For the second staining, cells were incubated in brilliant violet staining buffer (Becton, Dickinson [BD], San Jose, CA) and stained with the appropriate concentrations of surface antibodies for 20 min at 4°C (for a complete list of antibodies, see Table 1). Fluorescence-minus-one (FMO) staining control tubes were set up with all antibodies used in the experiment except for the measured one. FMO controls were set up for each antibody tested using the same sample as the one for the experimental group each time. Cells were then washed twice in PBS with 0.01% sodium azide (Sigma). Next, for intracellular staining, cells were incubated with either BD Cytofix/Cytoperm buffer, cytoplasmic staining buffer, or the eBioscience Foxp3/transcription factor staining buffer (Fisher) set (applied according to the manufacturer’s instructions) to fix and permeabilize cells. Fixed and permeabilized cells were then stained with antibodies to either cytokines or transcription factors.

**TABLE 1:** Antibodies used for flow cytometry and sorting

Antigen	Vendor	Clone
CD3	BDIS	SP34-2
CD8A	BDIS	53-6.7
CD20	BDIS	L27
CD45	BDIS	D058-1283
CD56	BDIS	NCAM 16.2
CD68	BDIS	Y1/82A
CD127	Beckman Coulter	R34.34
CD11c	BioLegend	3.9
CD122	BioLegend	TU27
CD123	BioLegend	6H6
CD34	BioLegend	581
CD303	BioLegend	201A
FasL	BioLegend	NOK-1
HLA-DR	BioLegend	L243
IFN-γ	BioLegend	4S.B3
Ki67	BioLegend	11F6
NKp44	Miltenyi	2.29
NKG2A/C	Beckman Coulter	HP-3B1
Granzyme A	BioLegend	CB9
Granzyme B	BioLegend	QA16A02
Granzyme K	Affymetrix	G3H69
TNF-α	BioLegend	MAb11
IgA	Rockland	Polyclonal
IL-18	R&D Systems	Polyclonal
Donkey anti-goat	Jackson	AF647
IgG	BDIS	G18-145
CD16	BioLegend	3G8
Perforin	MabTech	Pf-344
IgM	BDIS	G20-127
IL-18R	BioLegend	H44
Ig kappa	BioLegend	MHK-49
Ig lambda	BioLegend	MHL-38
CD21	Beckman Coulter	BL13
IL-1β	Affymetrix	CRM56
CD274	BioLegend	29E.2A3
CD185	BDIS	BB515
CD80	BioLegend	L307.4
CD32	BDIS	FL18.26
CD79a	BDIS	HM47
CD79b	BioLegend	CB3-1
CD86	BDIS	FUN-1
CD69	BioLegend	FN50
CD40	BDIS	5C3
NKp46	R&D Systems	BAB281
NKG2D	BioLegend	1D11
41BB	BioLegend	4B4-1

In experiments testing the nonspecific binding of fluorochrome-conjugated antibodies to CD16, cells were treated with unlabeled anti-CD16 or isotype control antibodies (30 min at 4°C) before adding fluorochrome-conjugated anti-CD16 antibodies (clones of fluorochrome-conjugated antibodies are the same as those for the unlabeled antibodies).

Our study used multicolor flow cytometry to detect NK cells, B cells, and NKB cells. Stained cells and their controls were collected, and data were processed on a BD LSRFortessa system. Data were analyzed using FlowJo software version 10.15 (FlowJo LLC). The thresholds for the detection of each cell type detection were >1,000 events for NK cells in SIV-infected macaques, >2,000 events for NKB cells in SIV-infected macaques and uninfected macaques, and >5,000 events for T cells and B cells.

Our study identified NK cells in the colons of SIV-infected macaques using widely accepted markers to identify NK cells in human mucosal tissues (70). Using FMO staining controls, we first gated on CD45^+^ cells and then determined the frequencies of NK cells among lineage-negative (Lin^−^) (i.e., CD3^−^, CD20^−^, CD11c^−^, CD34^−^, CD68^−^, CD123^−^, CD303^−^, and FcεR^−^) viable single cells (Fig. 1). It should be noted that CD68 is a marker that identifies monocytes and macrophages (71). Our studies shown in Fig. 1 to 4 identified NK cells as CD56^+^ CD127^−^ Lin^−^ CD56^+^. In the studies that we show in Fig. 5 to 9, we changed the gating to consider NK cells without CD56, which are within the NKG2A/C^+^ CD8α^+^ CD20^−^ CD3^−^ cells. We broke down the NK cells into subsets based on the differential expression of CD56 and CD16 (i.e., CD56^+^ CD16^−^, CD56^+^ CD16^+^, CD56^−^ CD16^+^, and CD56^−^ CD16^−^).

### Cytotoxicity assay.

A CytoTox 96-well nonradioactive assay (Promega, Madison, WI) was used to measure specific lysis via lactate dehydrogenase (LDH) release according to the manufacturer’s instructions. Plates were read on a Cytation 3 imaging reader (BioTek, Winooski, VT). NKB cells and B cells were fluorescence-activated cell (FAC) sorted with abort rates of >89% from thawed cryopreserved lamina propria cells stained with fluorochrome-conjugated antibodies (as shown in Fig. 1A) prior to the assay, with a purity of >99%. NKB cells were cultured at a 2:1 effector cell-to-target cell ratio of NKB or B cells to K562 target cells (NK cell-sensitive cells from the American Type Culture Collection). Assays of all groups were performed in triplicate.

### RNAseq and bioinformatic analyses.

The cells of the lamina propria of three SIV-positive and three uninfected healthy macaques were sorted to isolate NK, B, and NKB cells. Sorting was set for high stringency with low recovery. The abortive rate was >89%. All sorted samples were ≥99% positive. First, cells were gated on CD45^+^ and for singles. After this, live cells were selected, while CD3^+^ T cells were selected. Finally, cells were selected for CD20 and CD56 expression. NK cells were CD56^+^ and CD20^−^, B cells were CD20^+^ and CD56^−^, and NKB cells were CD56^+^ and CD20^+^.

RNA was isolated using a Qiagen RNeasy kit, and purified RNA was provided to the University of Illinois at Chicago Genome Research Core for RNAseq analysis. Sequencing libraries were prepared using a strand-specific QuantSeq 3′ RNAseq kit (Fwd, catalog no. 015; Lexogen). The QuantSeq protocol generates only 1 fragment per transcript, resulting in accurate gene expression values, and the sequences obtained are close to the 3′ ends of the transcripts. Total RNA at 10 ng per sample was used as an input. Library construction was performed according to the manufacturer’s protocol, with all modifications recommended for samples with a low RNA input.

In brief, during first-strand cDNA synthesis, an oligo(dT) primer containing an Illumina-compatible sequence at its 5′ end was hybridized to mRNA, and reverse transcription was performed. After that, the RNA template was degraded, and during second-strand synthesis, the library was converted to double-stranded DNA (dsDNA). Second-strand synthesis was initiated by a random primer containing an Illumina-compatible linker sequence at its 5′ end. The double-stranded libraries were purified using magnetic beads to remove all reaction mixture components. Next, the libraries were amplified to add the complete adapter sequences required for cluster generation and to generate sufficient quality control and sequencing material. The number of PCR amplification cycles was 22, as determined by quantitative PCR (qPCR) using a small preamplification library aliquot for each sample. The final amplified libraries were purified and quantified, and fragment sizes were confirmed to be within 263 to 318 bp by gel electrophoresis using an Agilent 4200 TapeStation (D1000 Screen Tape). The concentration of the final library pool was confirmed by qPCR. Sequencing was performed on the NextSeq 500 platform (Illumina), a high-output kit, with 1- by 75-nucleotide single reads. Raw reads were aligned to the reference genome mmul8 using BWA MEM (72). Ensembl gene expression levels were quantified using FeatureCounts (73). Differential expression statistics (fold change and *P* value) were computed using EdgeR (74, 75), using the generalized linear model framework to model variance associated with animal identifier while testing for cell type differences and correcting for animal-specific differences using the RemoveBatchEffect function. *P* values were adjusted for multiple testing using the false discovery rate (FDR) correction (76).

### Statistics.

Data, which are not part of the RNAseq data, were compiled and analyzed using GraphPad Prism 8 (version 8.3.1). Samples were compared using the Wilcoxon matched-pairs signed-rank test within each group.
